# Residue management alters microbial diversity and activity without affecting their community composition in black soil, Northeast China

**DOI:** 10.7717/peerj.5754

**Published:** 2018-10-10

**Authors:** Siyu Gu, Xingjun Guo, Yuetong Cai, Zehui Zhang, Shuai Wu, Xin Li, Huihui Zhang, Wei Yang

**Affiliations:** College of Resources and Environment, Northeast Agricultural University, Harbin, China

**Keywords:** Black soil, Residue management, Cow manure, Microbial community, Biolog Eco-Plates

## Abstract

Residue management is an important agricultural practice for improving soil fertility. To reveal the impact of residue management on soil microbial community, we conducted a field experiment with three treatments: no straw returning (control, CK), straw returning (SR), and straw returning combined with cow manure (SM). Our results indicated that soil organic matter content was significantly higher in SR treatment than CK in both seedling and jointing stages. In seedling stage, the lowest total nitrogen content was observed in CK treatment, and significantly lower than that in SM and SR treatment. Furthermore, soil available phosphorus content was significantly higher in SM and SR treatment than CK in jointing stage. In the seedling stage, the soil microbial average wellcolor development (AWCD) value, microbial McIntosh index, and Shannon index of CK and SM treatments were significantly higher than those in SR treatment. The AWCD value and McIntosh index in the jointing stage showed similar patterns: SM > CK > SR. Permutational multivariate analysis of variance indicated that soil microbial community was significantly affected by growth stage, but unaffected by residue management. The partial Mantel test revealed that the available potassium and the C/N ratio had independent effects on soil microbial community. Overall, our results indicated that straw returning combined with cow manure had a beneficial effect on soil fertility, microbial activity and diversity.

## Introduction

Black soil, which characterized by the highest fertility is one of the most important soil source in China (Giguere et al., 2017). Therefore, the black soil region in Northeastern China is important for producing commodity grains (Yin et al., 2015). However, serious soil erosion and fertility deterioration has occurred in this region due to irresponsible land management over the past few years (Liu et al., 2010), which severely threatens the grain production capability and ecological security of this region (Ou et al., 2017).

Straw returning is an effective strategy for organic waste recycling and beneficial practice for improving soil fertility. It ameliorates soil mainly through improving soil nutrient content and modifying soil microbial community (Zhao et al., 2018). The northeastern region of China is characterized by high latitude, resulting in cold winter and often summer droughts, which leads to slow straw decomposition, severely influencing sowing quality of next growing season. Therefore, traditional straw returning is not effective for practical agricultural production (Lv, 2006). Because of concerns about the soil degradation, many regions have adopted the method of combining straw returning with organic fertilizer to promote soil fertility.

In addition to straw returning, application of organic fertilizers can improve soil physical and chemical properties (Wang et al., 2017), increase nutrient utilization efficiency (Zhao et al., 2011), enhance soil microbial diversity (Gattinger et al., 2013; Sradnick et al., 2013), as well as depress soil-born pathogens (Zaccardelli et al., 2013). Therefore, amendment with organic fertilizer is important for the prevention of black soil degradation, improvement of crop yields (Jackson et al., 2012) and acceleration of the recycling of organic wastes. Soil microbes are important components of soil ecosystem (Pankhurst et al., 1996), which not only promote soil nutrient cycles by mineralization of soil organic matters, but also provide metabolites which act as nutrients for the plants (Kibblewhite, Ritz & Swift, 2008). Soil microbes are extremely sensitive to environmental variations so they can serve as biological indicators for soil quality and fertility (Lovell, Jarvis & Bardgett, 1995; Insam, Hutchinson & Reber, 1996; Steenwerth et al., 2003). Previous studies have shown that straw returning is beneficial for maintaining soil microbial diversity and activity (Chen et al., 2017). For instance, Zhao et al. (2016) reported that 30-year straw returning modified soil microbial community composition and increased the microbial activities through phospholipid fatty acid analysis in north-central China. Wang et al. (2012) found that straw returning for over 3 years can significantly promote microbial biomass in black soil, North China. Even though, neutral or negative effect of straw returning on soil properties was still reported (Liu et al., 2008; Yevdokimov et al., 2008). For instance, straw returning has no apparent impact on soil microbial communities in one-year experiment in clay loam soils, South China (Tan et al., 2010). Hence, it is necessary to carry out short-term experimental studies to elucidate the response of soil microbial communities to straw returning.

The present study aimed to determine the effects of short-term residue management on the soil fertility and the microbial community during the maize growing season in black soil, China. The aim of the present study was to (1) determine whether straw returning combined with cow manure would have more beneficial effects on soil fertility, microbial activity and diversity than straw returning alone; (2) explore the key soil variables in shaping soil microbial communities during straw degradation; (3) determine whether there is different response of soil microbial community to straw returning between the seedling and jointing stage.

## Materials and Methods

### Study area

The field experiment was carried out in Shuangcheng, Central part of Songnen Plain (45°45′N, 126°55′E). The soil at this study site is a typical black soil (classified as Mollisols, according to USDA soil taxonomy) with soil texture of clay loam. This region has a typical monsoon climate, with annual average temperature of about 4.4 °C, and the annual mean precipitation of 481 mm. The basic soil characteristics of arable layer were as follows: soil organic matter (SOM) content of 34.96 g kg^−1^, total nitrogen (TN) content of 1.51 g kg^−1^, total phosphorous (TP) content of 0.60 g kg^−1^, available phosphorous (AP) content of 53.69 mg kg^−1^, available potassium (AK) content of 0.23 g kg^−1^, slowly available potassium (RK) content of 0.86 g kg^−1^, and pH of 5.74.

### Experiment design

The field trial plots were established in 2015 with three types of residue management: (1) no straw returning (control, CK); (2) straw returning (SR), where the straw was ground (5 cm) and then returned to the soil after the maize was harvested with application rate of 0.5 t/ha; (3) straw returning combined with cow manure (SM), where the straw application rate was the same with treatment SR and cow manure application rate was 15 t/ha. Straw and cow manure were incorporated into soils together with tillage in autumn of 2015. There were three replicates for each treatment, resulting in nine plots (plot size: 6 m ×10 m each and 2 m separation from each other). The detailed distribution patterns of the plots were provided in Fig. A1. The cow manure used in present study was solid manure with soil organic carbon of 198.8 g/kg, TN of 17.5 g/kg and pH of 7.2. The test crop was maize (*Zea Mays* L.), genotype Hongshuo 616. Maize was planted at a density of 45,000 plants hm^−2^, and was sowed in early May. The field was ploughed to the depth of 30 cm after harvest. Chemical fertilizers were applied during seed sowing (N: 117 kg/ha, P: 23.58 kg/ha, K: 26.14 kg/ha) and at mid-tillering (N: 139.2 kg/ha).

### Soil sampling

Soil samples were collected in June (seedling stage) and July (jointing stage) of 2016. Briefly, nine soil cores were collected (0–20 cm) in each plot, and then mixed into one sample (ca. 2 kg) and stored in ice box and transported to laboratory for sample processing. Fresh soil samples were divided into two parts: one part was air dried, passed through 1 mm and 0.25 mm sieves, respectively, then stored at room temperature to measure physical and chemical properties; the other part was passed through a 1 mm sieve and stored at 4 °C for Biolog Eco-Plates™ analysis.

### Soil physicochemical variables

SOM was measured using a potassium dichromate-external heating method. TN was measured with the semi-micro Kjeldahl method. TP was digested with the perchloric acid-sulfuric acid method, and then measured with the Mo-Sb colorimetry method. AP was extracted with the 0.5 mol L^−1^ NaHCO_3_ and then measured with Mo-Sb colorimetrymethod. AK was measured with the 1 mol L^−1^ ammonium acetate extraction-flame spectrometry method. RK was measured with the 1 mol L^−1^ hot nitric acid extraction- flame spectrometry method. Soil pH was measured with the potentiometer method, based on water and soil ratio of 2.5:1 (Bao, 2000).

### Biolog Eco-Plates™ analysis

Biolog Eco-Plates™ analysis was used to characterize the soil microbial community according to Classen et al. (2003). Biolog Eco-Plates™ is based on tetrazolium dye reduction as an indicator of sole-carbon-source utilization, and has been applied to ecological studies to estimate metabolic potential of microbial communities (Insam & Rangger, 1997). Each Biolog EcoPlate™ consist of 96 wells containing 31 carbon sources and water as control, with each replicated three times. Fresh soil (equivalent to 10 g dried mass) was put into a 150 ml conical flask, and 90 ml of sterilized 0.85% physiological saline water was added. The mixture was then vortexed for one min and incubated in ice water bath for one min, which was repeated three times and then the solution was allowed to settle for 30 min. The supernatant was isolated and then diluted 103 fold in a soil suspension liquid, then 150 l of it was added to each pore of the Biolog ECO-plate. The inoculated plates were then placed into a 25 °C incubator and cultured for 216 h. Then the BioTek plate reader (Sunrise Remote; TECAN, Männedorf, Switzerland) was used to measure the absorbance at 590 nm every 24 h (Schutter & Dick, 2001).

### Data analysis

Absorbance value obtained from Eco-plate reader was subtracted by the absorbance at 0 h to remove background interference (Garau et al., 2007). Negative values were all set to zero. The average well color development (AWCD) was calculated at each culturing time, Shannon, McIntosh, and Simpson indices were all calculated based on the absorbance at 96 h (Wei et al., 2011).

The calculation formula for average well color development was as follows: AWCD $=\sum \left( Ci-R \right) /n$, where Ci is the optical density of each pore with culture medium; R denoted the optical density of the control pores; and n is the carbon source type of culture medium (which was 31 in this study). The calculation formulae for McIntosh, Shannon, and Simpson indices were as follows: McIntosh index(U): $U= \left( N-\sqrt{\sum \mathrm{n}i} \right) / \left( N-\sqrt{\mathrm{N}} \right) $; Shannon index (*H*′): ${H}^{{^{\prime}}}=-\sum \left( \mathrm{Pi}\cdot \log \mathrm{Pi} \right) $; Simpson index (D): *D* = 1 − ∑*Pi*^2^. In which: “Pi” is the ratio of the activity on a particular substrate to the sum of activities on all substrates, “N” refers to the relative absorbance value for each one of the C source wells by subtracting the absorbance value of the control well and, “ni” is the relative absorbance of well (Staddon, Duchesne & Trevors, 1997).

One-way ANOVA was used to examine the effects of residue management on soil microbial Shannon, McIntosh, and Simpson indices, total AWCD values, as well as the AWCD values of carbohydrates, amino acids, carboxylic acid, polymers, phenols, and ammonia at the maize seedling and jointing stages. All data were tested for normality and homogeneity of variance before ANOVA. Differences among treatments were tested by a Tukey’s HSD post-hoc test at *P* < 0.05. Pearson correlation analysis was used to determine the correlation between soil nutrients and Shannon, McIntosh and Simpson indices. Analysis above was carried out in SPSS 19.0 software. The vegan software package was used to calculate Shannon, McIntosh, and Simpson indices (Oksanen et al., 2013). The Permutational multivariate analysis of variance (PERMANOVA) using distance matrices was carried out in the vegan package (Oksanen et al., 2013) to evaluate the effects of residue management, growth stage, and their interaction on soil microbial community composition. Ecodist software package was employed to conduct Mantel and partial Mantel tests (Goslee & Urban, 2007). Moreover, the ‘varpart’ function in the vegan package was used to partition the variation of soil microbial community dissimilarity by residue management, growth stage, and soil variables (SOM, TN, TP, AP, AK, RK, pH). Analysis above was conducted with R 1.3.3 (R Core Team, 2013).

## Results

### Effects of residue management on soil nutrient contents

**Figure 1:**
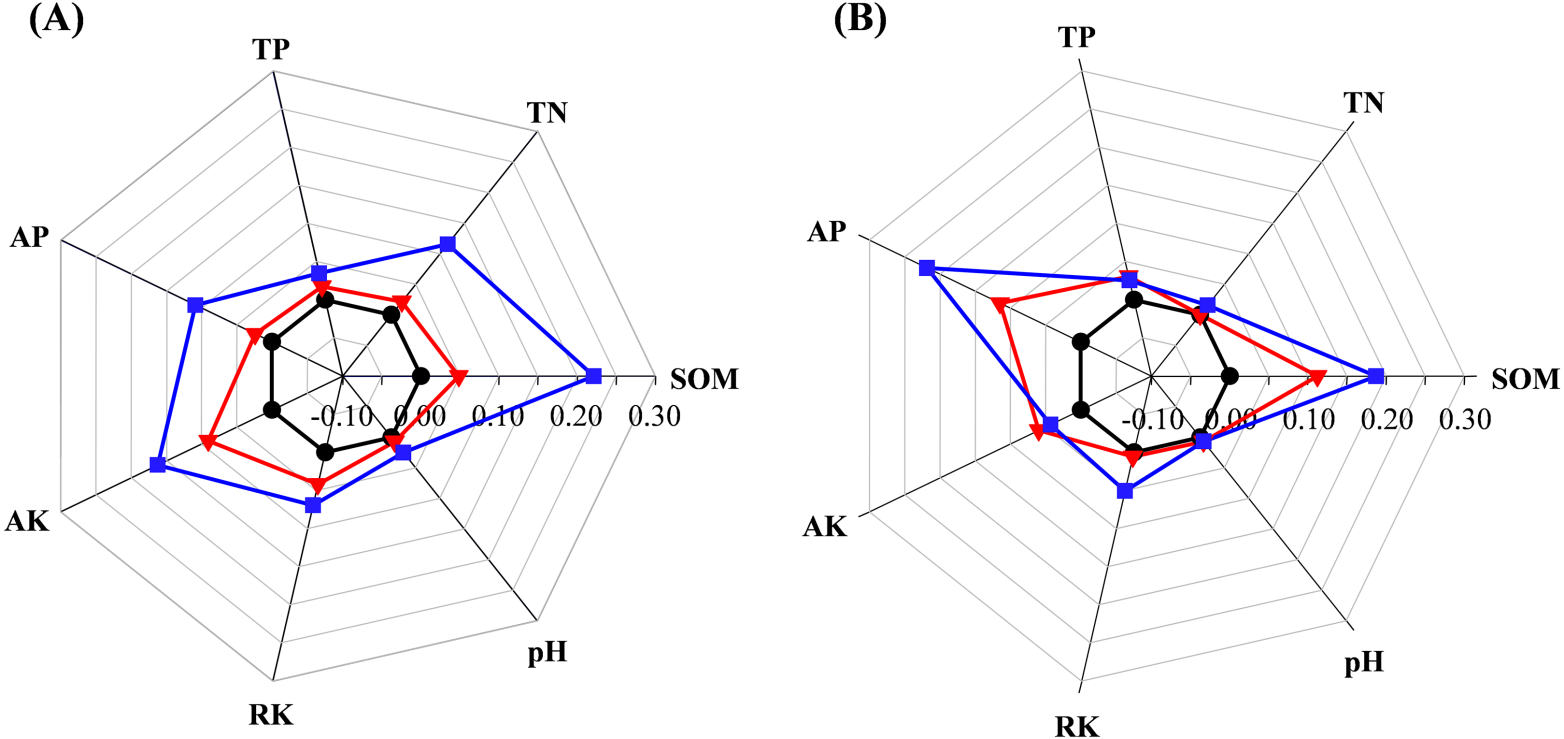
Percentage of soil organic matter (SOM), total nitrogen (TN), total phosphorus (TP), available phosphorus (AP), available potassium (AK), slow available potassium (RK) and pH compared to control (=0%) in the maize seedling (A) and the jointing (B) stage. Black, control; red, straw returning; blue, straw returning combined with cow manure.

ANOVA analysis showed that residue management significantly affected TN content in seedling stage and AP content in jointing stage (Table 1). SOM content was significantly higher in SM treatment than CK in both the seedling and jointing stages (Fig. 1, Table S1). In the seedling stage, the lowest TN content was observed in CK treatment, and significantly lower than that in SM and SR treatments (Fig. 1A, Table S1). Furthermore, AP content was significantly higher in SM and SR treatments than CK in the jointing stage (Fig. 1B, Table S1).

**Table 1:** One-way ANOVA examining the effects of residue management on soil variables.

	Seedling		Jointing	
Variables	*F*	*P*	*F*	*P*
SOM	5.76	**0.04**	3.40	0.10
TN	44.08	**<0.01**	0.14	0.87
TP	0.30	0.75	0.60	0.58
AP	1.17	0.37	17.09	**<0.01**
AK	0.23	0.80	0.11	0.90
RK	0.34	0.73	1.32	0.33
pH	0.76	0.51	1.53	0.29
AWCD	16.93	**<0.01**	9.43	**0.01**
Carbohydrates	14.25	**0.01**	37.46	**<0.01**
Polymers	0.97	0.43	6.38	**0.03**
Amino acids	2.48	0.16	4.99	**0.05**
Carboxylic acids	1.99	0.28	10.55	**0.01**
Phenols	2.73	0.14	1.01	0.42
Amines	28.06	**<0.01**	6.62	**0.03**

**Notes.**

Abbreviations SOM organic matter TN total nitrogen TP total phosphorus AP available phosphorus AK available potassium RK slow available potassium. AWCD average well color development

Values at *P* < 0.05 are shown in bold.

### The color development of soil microbial community

**Figure 2:**
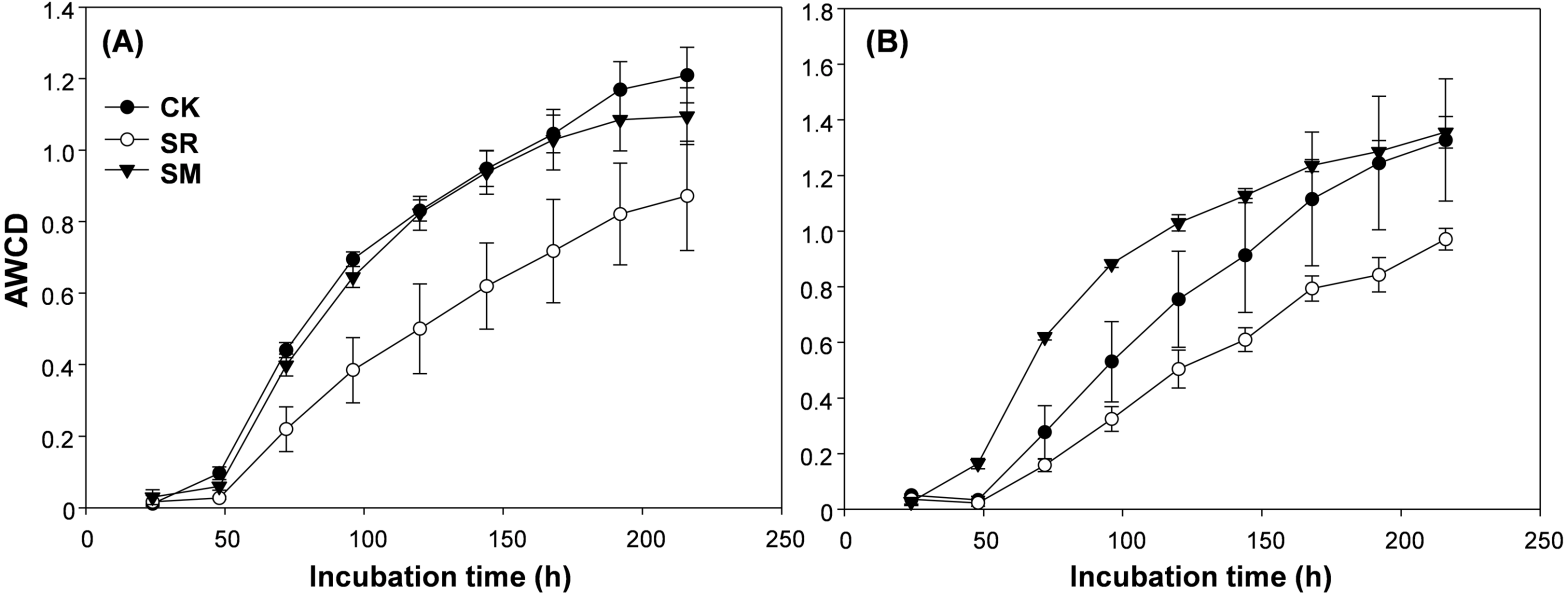
Average well color development (AWCD) in the maize seedling (A) and the jointing (B) stage across the incubation time. Abbreviations: CK, control; SR, straw returning; SM, straw returning combined with cow manure.

**Figure 3:**
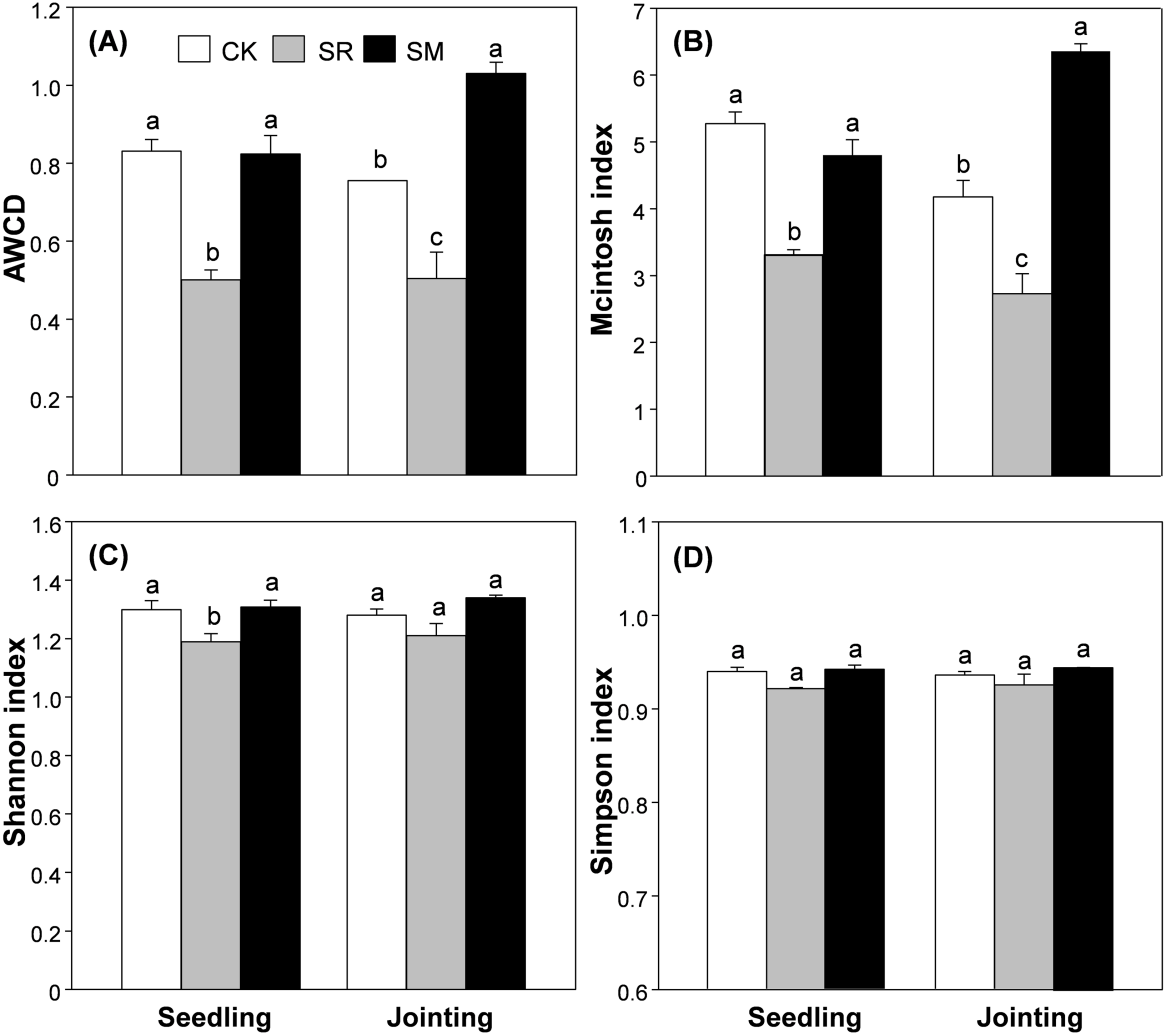
Average well color development (A), McIntosh (B), Shannon (C) and Simpson (D) indices among treatments. Abbreviations: CK, control; SR, straw returning; SM, straw returning combined with cow manure. Bars without shared letters indicate significant difference at *P* < 0.05.

AWCD reflected the soil microbial community’s carbon source utilization and metabolic activity. As shown in Fig. 2, soil microbial metabolic activity in both the seedling and jointing stages were relatively low in the first 48 h, but after this lag phase, the values increased greatly, After 192 h, the AWCD of all three treatments reached plateau. Residue management significantly influenced the AWCD at 96 h in both the seedling and jointing stages (Table 1). In the seedling stage, the AWCD of CK and SM treatments were significantly higher than the SR treatment (Fig. 3A). In the jointing stage, the AWCD was significantly different among treatments and showed the following pattern: SM > CK > SR (Fig. 3B).

### Changes in soil microbial functional diversity

Pearson correlation analysis showed that both Simpson and Shannon indices were positively correlated with AP content (Fig. A1). In the seedling stage, the McIntosh and Shannon indices were significantly higher in CK and SM treatments than in the SR treatment (Figs. 3B 3C). However, the Simpson index did not differ significantly among the three treatments (Fig. 3D). In the jointing stage, McIntosh index was significantly lower in the SM treatment than the other treatments, while the Simpson and Shannon indices were not obviously different among treatments.

### Utilization of six categories of carbon sources

**Figure 4:**
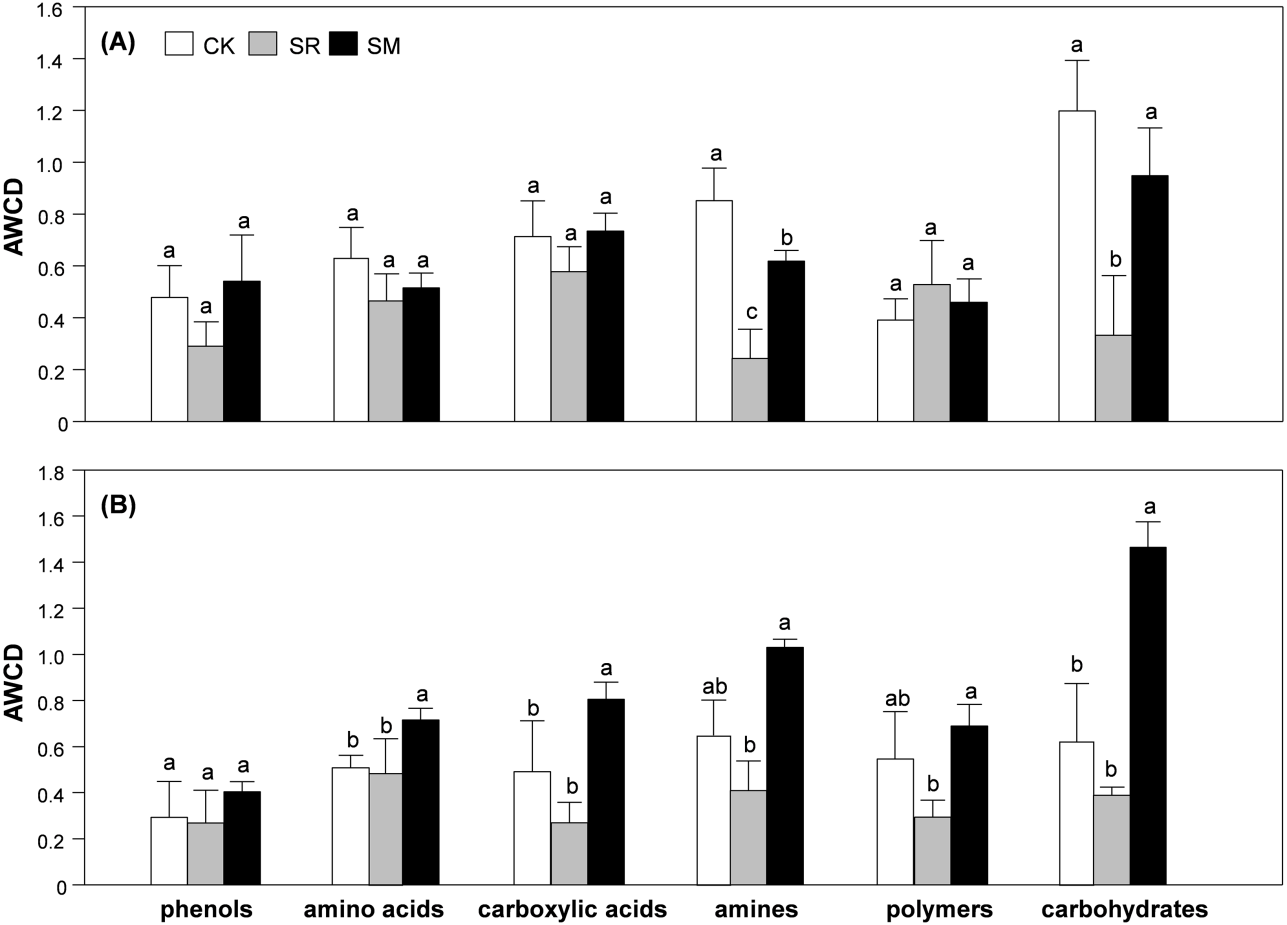
Average well color development (AWCD) for phenols, amino acids, carboxylic acids, amines, polymers and carbohydrates in maize at the seedling (A) and the jointing (B) stage. Abbreviations: CK, control; SR, straw returning; SM, straw returning combined with cow manure. Bars without shared letters indicate significant differences at *P* < 0.05.

In the seedling stage, the AWCD values of carbohydrates and amines were significantly affected by residue management (Table 1). Both AWCD of carbohydrates and polymers were lowest in SR treatment, and significantly lower than that in treatment CK and SM (Fig. 4A). In the jointing stage, AWCD of all carbon sources (except phenols) were significantly different among treatments. The SM treatment exhibited the highest AWCD value of all six categories of carbon sources. Among them, the AWCD value of amino acids, carbohydrates and carboxylic acids in treatment SM was significantly higher than CK and SR treatment, whereas the AWCD value of amines and polymers in treatment SM only differed with treatment SR (Fig. 4B).

### Soil microbial community structure

**Figure 5:**
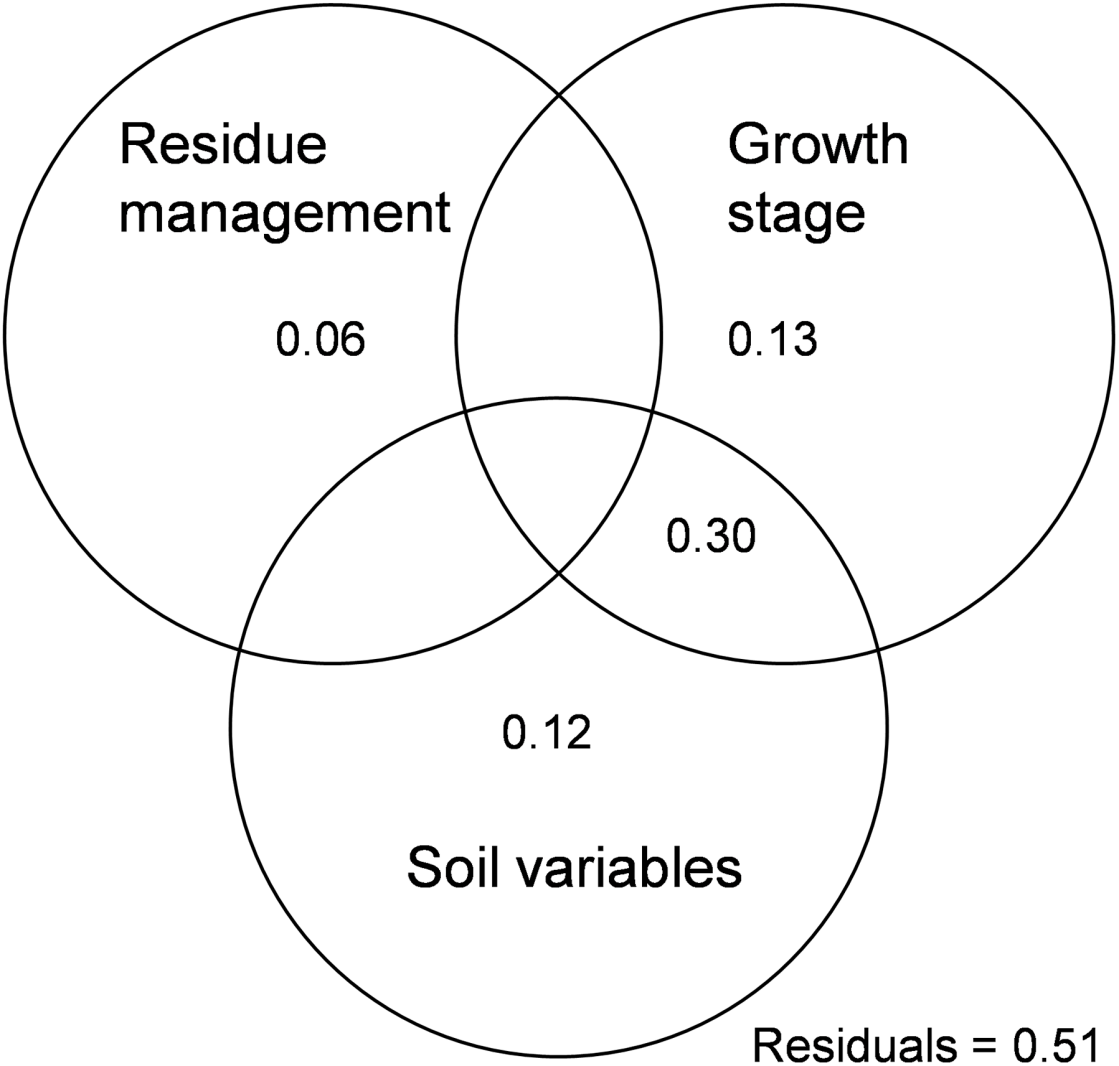
Variation partitioning analysis showing the effects of residue management, growth stages and soil variables on soil microbial community. Numbers inside circles indicate the proportion of explained variation. Soil variables include SOM, TN, TP, AP, AK, RK and pH.

PERMANOVA analysis indicated that soil microbial community was significantly affected by sampling time (*r*^2^ = 0.68, *P* < 0.01), but unaffected by residue management (*r*^2^ = 0.01, *P* = 0.95) and their interaction (*r*^2^ = 0.01, *P* = 0.94). Additionally, variation portioning analysis indicated that 49% of soil microbial community composition was explained. Among these variations, 6% was explained by residue management, 43% by sampling time, and 42% by soil variables (Fig. 5). Mantel test analysis indicated that soil microbial community structure was significantly influenced by SOM, TN, AK, and C/N, among which AK and C/N showed independent effects on microbial community (Table 2).

**Table 2:** Mantel and partial Mantel tests of the soil microbial community with soil variables.

	Mantel test		Partial mantel test	
	*r*	*P*	*r*	*P*
SOM	0.41	**<0.01**	−0.07	0.82
TN	0.28	**0.01**	0.13	0.06
TP	−0.03	0.65	0.02	0.32
AP	−0.06	0.86	−0.08	0.90
AK	0.27	**0.01**	0.31	**<0.01**
RK	0.12	0.07	<0.01	0.40
pH	0.09	0.07	0.06	0.15
C/N	0.65	**<0.01**	0.51	**<0.01**

**Notes.**

Abbreviations SOM organic matter TN total nitrogen TP total phosphorus AP available phosphorus AK available potassium RK slow available potassium

Values at *P* < 0.05 are shown in bold.

## Discussion

It was widely reported that SOM content was significantly enhanced after long term straw returning (Zhang, Liu & Li, 2009; Moharana et al., 2012). Our results indicated that the SR treatment only induced a slight increase in SOM content. This result was mainly attributed to the geographical position and the climatic conditions in present study. The northeastern region in China was characterized by high latitude and water and heat deficiencies, which lead to the slow decomposition of the straw (Gao et al., 2013). Furthermore, soil nitrogen was highly related to organic carbon content, and their mineralization and priming effect had obvious positive interactions (Balesdent et al., 1998), hence the TN content also significantly increased in the treatment SM. Previous studies showed that the long-term application of chemical fertilizers induced reduction in soil pH (Guo et al., 2010) but applying organic fertilizers could ameliorate the acidity of soil (Yang et al., 2017) and release a large amount of phosphate ions (Liang et al., 2012). Therefore, straw returning combined with cow manure could significantly increase AP content. Taken as a whole, our results indicated that straw returning combined with cow manure significantly increased the amount of SOM, TN, and AP compared with control, which was consistent with the previous studies (Zhang, Liu & Li, 2009; Moharana et al., 2012; Cheng et al., 2014). However, we found that straw returning did not significantly affect TP, AK, and RK content, which might have been due to the relatively short experiment duration (Bernard et al., 2012).

AWCD is an important indicator reflecting carbon source utilization and the metabolic activity of the soil microbial community (Garland, 1997; Konopka, Oliver & Turco Jr, 1998) This study showed that straw returning alone inhibited soil microbial activity. Also, the SR treatment exhibited the lowest AWCD value of all six categories of carbon sources. This was mainly because the application of maize straw may increase C/N ratio in soil (Gunapala & Scow, 1998), which induced competition in microbes and crops for nitrogen and inhibited microbial activity (Yevdokimov et al., 2008). In the same way, Liu et al. (2008) found that microbial activity was inhibited at the early stages of straw returning in cotton field. However, straw returning combined with cow manure significantly enhanced soil microbial activity. This may be due to the cow manure amendment provides substrates such as carbohydrates, amino acid, amine compounds for soil microorganisms. Alternatively, the exogenous microbes introduced to soil from manure would be another reason (Sun et al., 2016).

Both Shannon and McinIosh indices were depressed in the SR treatment compared with the control, which might because the straw application interfered with the soil-plants-microbe balance (Li et al., 2015) and inhibited the growth of some microbial taxa. On the other hand, straw returning combined with cow manure did not cause significant shift in the Shannon diversity index. Peng et al. (2016) found that the effect of short-term straw returning on microbial community composition was limited in the ecosystem. In the same way, Scotti et al. (2016) failed to detect a change in the Shannon index of soil microbes after applying organic fertilizer for one year. However, Zhao et al. (2016) showed that the application of organic fertilizers for 30 years resulted in significant increases in soil microbial diversity. Moreover, soil microbial diversity was greatly enhanced after applying organic fertilizers 15 years in a long-term field experiment through Miseq sequencing (Hartmann et al., 2015). Allison & Martiny (2008) proposed that soil microbial community had high metabolic and physical tolerances to environment changes (Meyer et al., 2004), hence the microbial community would not change rapidly within a short period. Therefore, the lack of effect of straw returning on soil microbial community was possibly due to the duration of our experiment.

PERMANOVA analysis indicated that soil microbial community was unaffected by residue management. Moreover, plant growth stage was observed to be a significant factor in shaping soil microbial community in present study. Two mechanisms would be responsible for this result. Firstly, a large number of studies have shown that soil microbial community can be significantly influenced by soil physical and chemical properties (Fließbach et al., 2007; Cusack & Firestone, 2011; Song et al., 2017). In the present study, the AP content in soil was positively correlated with both the Shannon diversity and Simpson diversity indices. Tang et al. (2016) previously indicated that microbial activity was limited by the soil P availability. It was reported that phosphorous can control the growth and development of some phosphorous-sensitive bacteria and fungus, inducing changes in soil microbial community structure (Beauregard et al., 2010). As confirmed by the Mantel test, soil C/N ratio was also shown to have an independent effect on microbial community. Soil C/N ratio is an important indicator of soil organic carbon effectiveness (Yao et al., 2017). Previous studies have shown that soil oligotrophic bacteria will be enriched with high C/N ratio, while when the C/N ratio was lower, copiotrophic bacteria will be favored (Fierer, Bradford & Jackson, 2007). Therefore, the C/N ratio may induce changes in microbial community composition by affecting the growth of different microbial groups. Secondly, the shift in soil microbial communities may be attributed to the change in soil temperature and moisture (Siles et al., 2016).

Although Biolog Eco-Plates is a useful tool to explore the functional changes in microbial communities, it was argued that this method tends to be biased towards fast-growing bacteria rather than the slowing-growing fungi (Preston-Mafham, Boddy & Randerson, 2002). However, fungi are one of the most abundant microorganisms in soil and play crucial roles in straw degradation (Finlay, 2002). Therefore, future study is necessary to characterize both bacterial and fungal community compositions in response to residue management using high-throughput sequencing.

## Conclusion

In conclusion, our results demonstrated that SR treatment significantly decreased the microbial activity and diversity compared with control in all growth stages, while SM treatment maintained the microbial diversity and enhanced the microbial activity compared with control. PERMANOVA analysis indicated that the soil microbial community structure was affected by the growth stage rather than residue management. Soil AP and C/N ratio were the most important drivers for shaping soil microbial communities during maize straw decomposition. Overall, our results indicated that straw returning combined with cow manure had a beneficial effect on soil fertility, microbial activity and diversity.

##  Supplemental Information

10.7717/peerj.5754/supp-1 File S1 Raw data of soil physiochemical variables

10.7717/peerj.5754/supp-2 File S2 Raw data of Biolog Eco-plates analysis in seedling stage

10.7717/peerj.5754/supp-3 File S3 Raw data of Biolog Eco-plates analysis in jointing stage

10.7717/peerj.5754/supp-4 Supplemental Information 1 Distribution patterns of the plots in this study site Control: 1, 4, 7; straw returning: 2, 5, 8; straw returning combined with cow manure: 3, 6, 9.

10.7717/peerj.5754/supp-5 Supplemental Information 2 Relationship between soil available phosphorus (P) and soil microbial Simpson index (A) and Shannon index (B)

10.7717/peerj.5754/supp-6 Table S1 Soil organic matter (SOM), total nitrogen (TN), total phosphorus (TP), available phosphorus (AP), available potassium (AK), slow available potassium (RK) and pH among treatments

## Additional Information and Declarations

### Data Availability

The following information was supplied regarding data availability: The raw data are provided in the Supplemental Files.
