# Supplementary figures and images for: Residue management alters microbial diversity and activity without affecting their community composition in black soil, Northeast China
Source: PeerJ. 2018 Oct 10;6:e5754. doi: 10.7717/peerj.5754 (PMC6186157; doi:10.7717/peerj.5754)

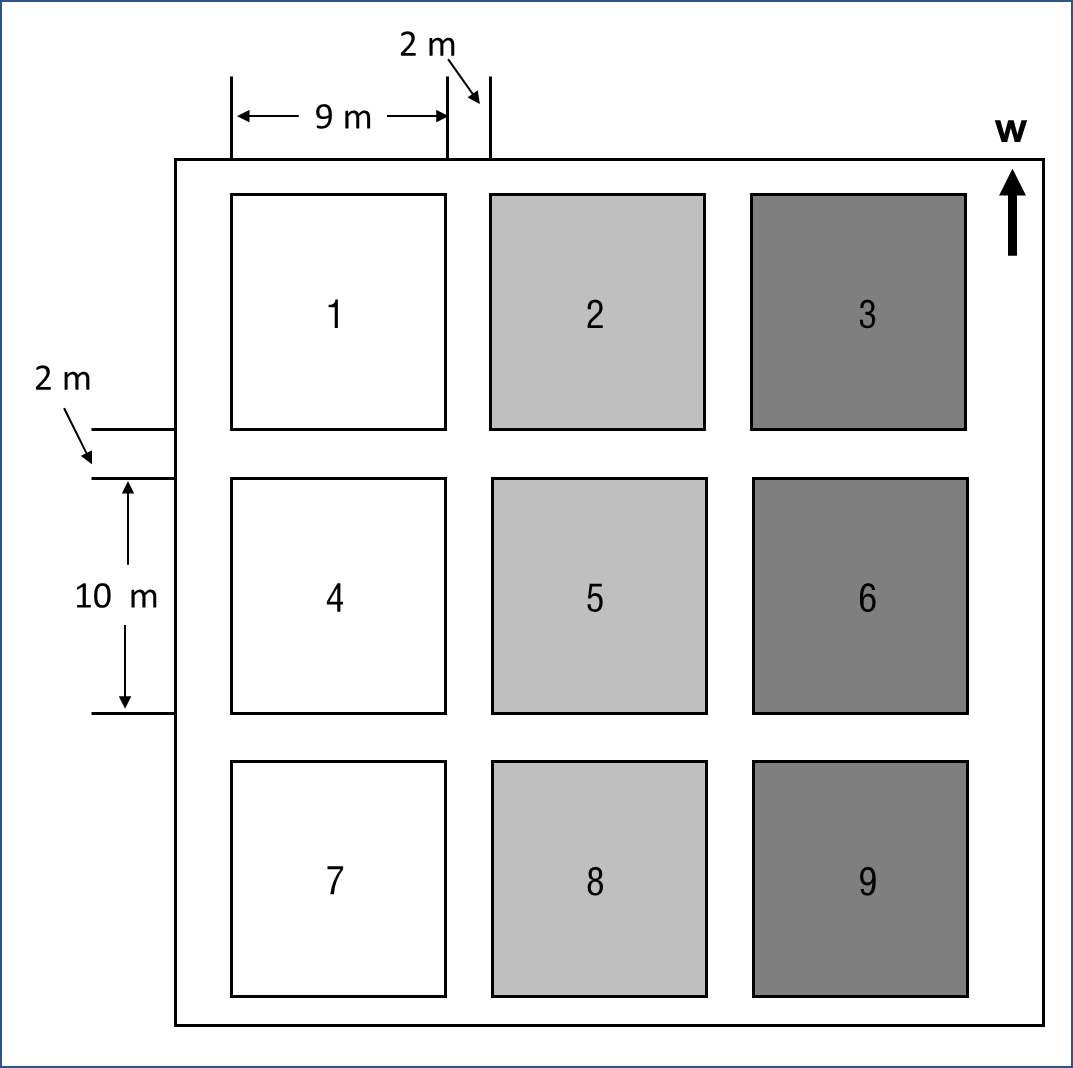

Supplement: Supplemental Information 1 — Control: 1, 4, 7; straw returning: 2, 5, 8; straw returning combined with cow manure: 3, 6, 9. [file peerj-06-5754-s004.png]

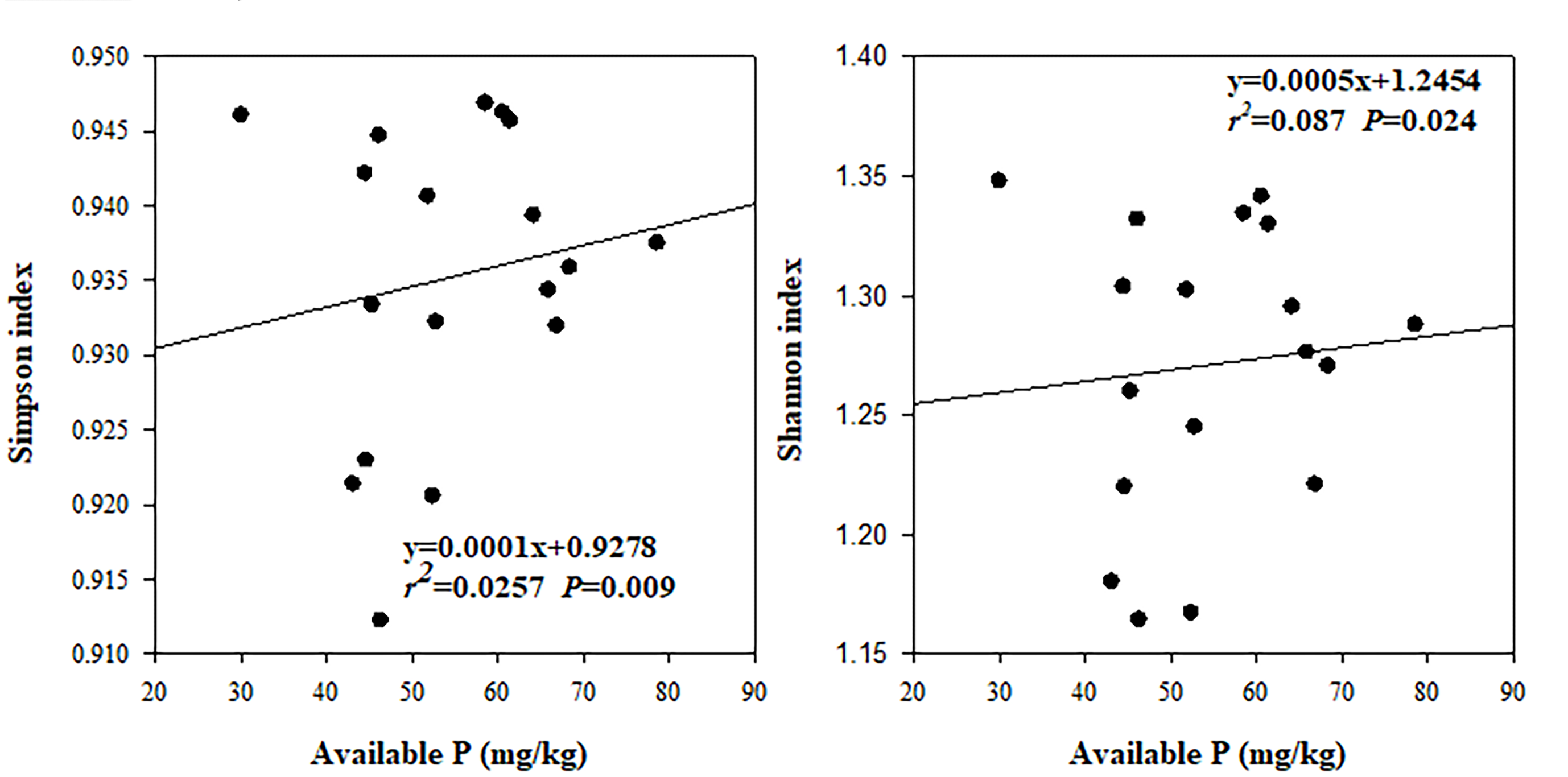

Supplement: Supplemental Information 2 [file peerj-06-5754-s005.png]
