# Supplementary material for: Residue management alters microbial diversity and activity without affecting their community composition in black soil, Northeast China
Source: PeerJ. 2018 Oct 10;6:e5754. doi: 10.7717/peerj.5754 (PMC6186157; doi:10.7717/peerj.5754)
Supplement: Table S1 [file peerj-06-5754-s006.docx]

| Table S1. Soil organic matter (SOM), total nitrogen (TN), total phosphorus (TP), available phosphorus (AP), available potassium (AK), slow available potassium (RK) and pH among treatments in seedling and jointing stage. | | | | | | | |
| --- | --- | --- | --- | --- | --- | --- | --- |
|  | Seedling stage | | |  | Jointing stage | | |
|  | CK | ST | SM |  | CK | ST | SM |
| SOM | 27.85+0.49 b | 29.85+2.17 ab | 33.96+3.19 a |  | 34.4+4 a | 38.25+2.74 a | 40.84+2.08 a |
| TN | 1.36+0.02 c | 1.45+0.03 b | 1.57+0.03 a |  | 1.3+0.13 a | 1.33+0.07 a | 1.35+0.12 a |
| TP | 0.58+0.06 a | 0.61+0.05 a | 0.6+0.04 a |  | 0.61+0.06 a | 0.6+0.01 a | 0.58+0.02 a |
| AP | 59.53+11.84 a | 46.67+4.99 a | 60.53+17.18 a |  | 48.65+1.81 b | 54.2+0.1 ab | 59.24+3.39 a |
| AK | 271.67+12.5 a | 281.33+15.04 a | 271+30.51 a |  | 233.33+14.5 a | 243+34.51 a | 235.33+25.77 a |
| RK | 855.84+18.32 a | 850.51+9.22 a | 878.19+73.22 a |  | 888+50.39 a | 893.33+17.16 a | 933+35.76 a |
| pH | 5.76+0.12 a | 5.73+0.04 a | 5.68+0.05 a |  | 5.53+0.19 a | 5.63+0.04 a | 5.72+0.12 a |
